# Acute Type A Aortic Dissection and Late Pregnancy: What Should We Do?

**DOI:** 10.21470/1678-9741-2020-0670

**Published:** 2023

**Authors:** Lingchao Liu, Chencheng Liu, Tianbo Li, Bo Xu, Yingbin Xiao, Yong Wang

**Affiliations:** 1 Institute of Cardiovascular Surgery, Xinqiao Hospital, Army Medical University, Chongqing, China.

**Keywords:** Aneurysm, Dissecting, Pregnancy, Chest Pain, Thorax, Follow-Up Studies, Treatment Outcome

## Abstract

**Introduction:**

Acute type A aortic dissection (AAAD) in late pregnancy is a rare but severe disease. Lack of clinical experience is the main cause of high mortality. This study tries to investigate the multidisciplinary therapeutic strategy for these patients.

**Case presentation:**

We reported three patients with AAAD in late pregnancy. Sudden chest pain was the main clinical symptom before operation. All three patients and their newborns survived through multidisciplinary approach in diagnosis and treatment. No serious complications occurred during the mid-term follow-up.

**Conclusion:**

Multidisciplinary diagnosis and treatment strategy play a crucial role in saving the lives of pregnant women with AAAD.

## INTRODUCTION

Acute type A aortic dissection (AAAD) is a life-threatening clinical emergency. The mortality of patients with this condition would rise at a rate of 1%-2% per hour approximately if not diagnosed and managed timely and properly, and nearly 50% of patients would die within a week^[1]^. Pregnancy complicated by aortic dissection is much more rarely encountered, accounting for only 0.1% of all patients with aortic dissection^[2]^. However, the risk of developing aortic dissection is much higher in pregnant women, since the aorta will be dilated due to increased cardiac and vascular volume caused by physical changes during pregnancy, such as fetoplacental circulation, enlarged uterus, endocrine changes, and increases in circulating blood volume. As reported, over 50% AAAD female patients under 40 years old suffered from this disease during pregnancy^[3]^. Therefore, pregnancy has been regarded as an independent risk factor for aortic dissection^[4]^. Due to the complex nature of pregnancy complicated by an associated AAAD, a multidisciplinary expert team should be involved to make every endeavor for the best outcomes for both the mother and the fetus. In this report, we presented three cases of late pregnancy complicated by AAAD that were managed by a multidisciplinary team (MDT) at our center.

### Case Presentation

#### Case 1

A 30-year-old female (*gravida 2, para 1*) at 34 weeks’ gestation presented to our emergency department with chest pain, weakness of right limb and blurred vision in her right eye for 6 hours in May 2018. No special history was found. Her mother, however, died from AAAD 3 years ago.

**Fig. 1:**
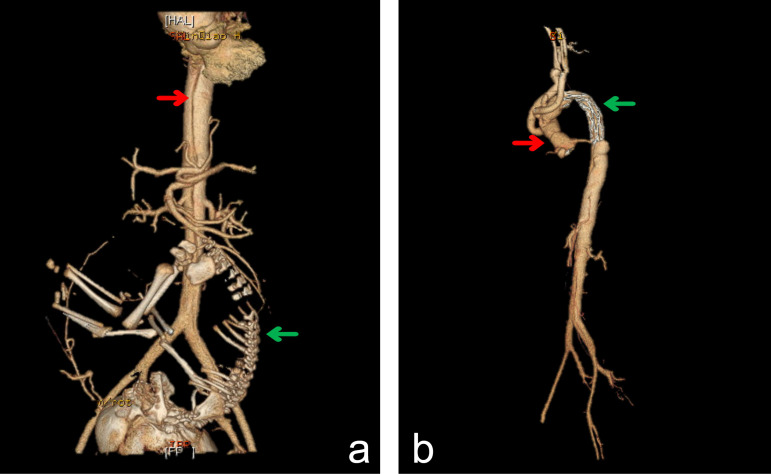
Changes in pre- and postoperative computed tomography angiography images of Case 1. (A) 3D reconstruction of preoperative CT images shows the location of thoracoabdominal aorta dissection marked by a red arrow and the fetal skeleton marked by a green arrow. (B) 3D reconstruction of postoperative CT images shows the artificial blood vessel marked by a red arrow and the covered stent graft marked by a green arrow.

Physical examination revealed disproportionately long extremities, slender fingers, and an cardiac function NYHA class III. In the meantime, the fetal heart rate was 140 beats per minute, revealing that there was no fetal distress. Echocardiography revealed aortic insufficiency (AI), an enlarged ascending aorta (internal diameter of 52 mm) with an aortic arch of normal size (internal diameter of 25 mm), intimal structure floating in the lumen of ascending aorta and aortic arch, and a defect about 59 mm away from the aortic valve. FS and LVEF were 40% and 70%, respectively. Three-dimensional reconstruction of computed tomography (CT) revealed that the dissection occurred from thoracic aorta to left common iliac artery and the aneurysmal dilation of the ascending aorta was formed (Figure 1A). Genetic test was positive for a *FBN1* mutation.

Based on these findings, a pregnancy complicated by acute aortic dissection (Stanford type A) and Marfan syndrome was suspected. Considering the complex and challenging situation, invasive monitoring was established for the well-being of the mother and the fetus after the patient’s arrival, and an MDT involving specialists from emergency, cardiovascular surgery, intensive care, anesthesia, pediatrics, obstetrics, echocardiography, radiology, genetic counseling, and patient communication unit was urgently orchestrated to ensure an optimal outcome. The patient and her family agreed with surgical repair of the aorta and cesarean delivery after thorough communication, and a written informed consent was obtained.

Therefore, 10.7 hours after arrival, the patient was escorted to operating, and an MDT was formed. After comprehensive multidisciplinary discussion, a decision was reached for a staged surgical intervention, which consisted of repeat C-section, intrauterine balloon tamponade and bilateral tubal ligation, since the baby was clearly viable and the patient, diagnosed with Marfan syndrome, had no intention for further pregnancy, followed by replacement of the aortic root with a mechanical valved conduit (Bentall procedure), total aortic arch replacement (TAR) and frozen elephant trunk (FET).

The patient was given intravenous remifentanil (1 µg/kg) and vecuronium bromide (0.8 mg/kg) as induction of anesthesia, then intravenous remifentanil (0.1 µg/kg/min) and inhalation of 2% sevoflurane and oxygen (2 L/min) as maintenance. The abdominal wall was incised longitudinally and a transverse incision was made in the lower uterine segment. The fetus was quickly delivered by the obstetric team, and the placenta was completely and naturally delivered. During repeat C-section, the patient was closely monitored by the echocardiographic team, while the baby was managed and monitored by the pediatric team immediately after delivery. Apgar scores of the newborn were 9, 10, and 10 at 1, 2 and 10 minutes, respectively. Meanwhile, intrauterine balloon tamponade, intravenous oxytocin (10 U) and carboprost tromethamine injection (250 µg) were given in prevention of postpartum hemorrhage. Subsequently, bilateral tubal ligation was performed and the abdomen was closed, thus allowing the cardiovascular surgery team to take over. Bentall procedure was performed under mild hypothermic cardiopulmonary bypass. After Bentall procedure, the body temperature was further decreased to facilitate TAR and FET, since the aortic arch and descending aorta were affected by acute aortic dissection (AAD). The bypass was stopped when nasopharyngeal and rectal temperatures were 24 ℃ and 26 ℃, respectively. Subsequently, the innominate artery, the left common carotid artery and the left subclavian artery were clamped after the patient was placed in an upside-down position. Selective antegrade cerebral perfusion via innominate artery was performed with cerebral oxygen saturation closely monitored through a transcutaneous oxygen monitor. TAR and FET were concurrently performed. Del Nido cardioplegic solution was administered during surgery to protect the myocardium. Heartbeat spontaneously returned after surgery. Operating time, cardiopulmonary bypass time, aortic clamping time and selective cerebral perfusion time were 10.4 hours, 273 minutes, 178 minutes and 32 minutes, respectively. The patient was transferred to the intensive care unit (ICU) for close monitoring immediately after surgery. She was successfully extubated 15.8 hours after surgery, and the balloon was deflated 33.8 hours after surgery.

She was transferred from ICU to the general ward 5 days later, where she developed hypoxemia, which was relieved by enhanced suctioning and oxygen therapy. She was discharged home 18 days later. To date, she is alive with an estimated cardiac function NYHA class I. The FS and LVEF during follow-up were 37% and 67%, respectively. Computed tomography angiography (CTA) revealed that the intraluminal stent was patent in the aortic arch and descending aorta while small aortic dissection was still located in the lower portion of abdominal aorta (Figure 1B). Meanwhile, the baby developed normally.

#### Case 2

A 32-year-old female (*gravida 2, para 1*) at 37 weeks’ gestation arrived at our emergency department with sudden onset nontraumatic chest and back pain for 6 hours in September 2018. No significant past medical history was stated, and family history revealed that one of her mother’s sisters was a confirmed victim of Marfan syndrome. The NYHA class was III.

**Fig. 2:**
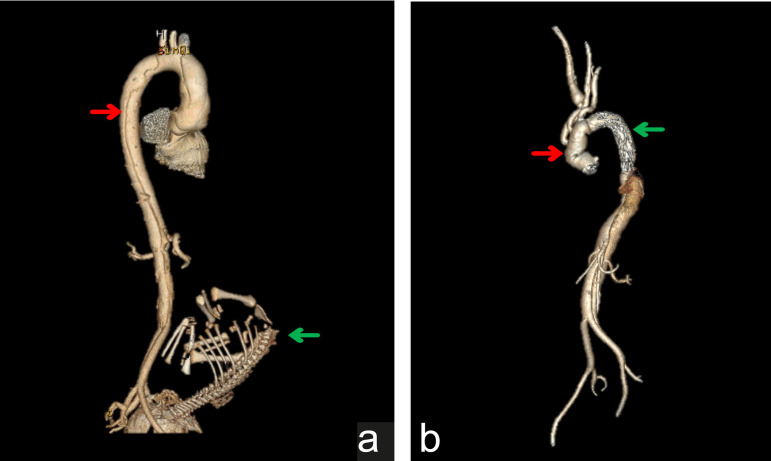
Changes in pre- and postoperative computed tomography angiography images of Case 2. (A) 3D reconstruction of preoperative CT images shows the location of thoracoabdominal aorta dissection marked by a red arrow and the fetal skeleton marked by a green arrow. (B) 3D reconstruction of postoperative CT images shows the artificial blood vessel marked by a red arrow and the covered stent graft marked by a green arrow.

In the meantime, no fetal distress occurred, as the fetal heart rate was 136 beats per minute. Echocardiography revealed AI, an enlarged ascending aorta (internal diameter of 39 mm), an enlarged aortic sinus (internal diameter of 41 mm), and intimal structure floating in the lumen of the ascending aorta and aortic arch. The FS and LVEF were 41% and 61%, respectively. CT scan revealed that the aortic dissection started from the aortic sinus to the proximal end of the bilateral common iliac artery, indicating a Stanford-A acute aortic dissection (Figure 2A). Considering the complex nature of a late pregnancy accompanied by AAD and suspected Marfan syndrome, invasive monitoring was established upon the patient’s arrival. The patient and her family agreed with surgical repair of aorta and cesarean delivery after thorough communication, and a written informed consent was obtained.

Subsequently, 5.1 hours after arrival, the patient was escorted to operating room, and an MDT was promptly formed. A staged surgical method was planned, which was similar to the plan in Case 1, except the bilateral tubal ligation since the patient and her family wanted to preserve fertility. The anesthetic and surgical procedures were similar to that of Case 1. Apgar scores of the newborn were 9, 10, and 10 at 1, 2 and 10 minutes, respectively. Heartbeat returned with the aid of electrical cardioversion after surgery since it did not return spontaneously. Operating time, cardiopulmonary bypass time, aortic clamping time and selective cerebral perfusion time were 9.1 hours, 234 minutes, 151 minutes and 35 minutes, respectively. The patient was transferred to the ICU for close monitoring immediately after surgery. The balloon was deflated 20.6 hours after surgery, and the patient was successfully extubated 20.7 hours after surgery. She was transferred from the ICU to general ward 3 days later, where she developed continuous hoarseness, caused by left recurrent laryngeal nerve paralysis. She was discharged home 15 days later. The patient developed left-sided spontaneous pneumothorax 48 days after surgery and left-sided hydropneumothorax 85 days after surgery, both relieved by closed thoracic drainage in our center. To date, she is alive with an estimated NYHA class II. The FS and LVEF during follow-up were 36.6% and 66.3%, respectively. CTA revealed that the dissection was still present in the descending aorta and abdominal aorta without progression (Figure 2B). Meanwhile, the baby was growing healthy.

#### Case 3

A 27-year-old female (*gravida 1, para 0*) at 37 weeks’ gestation arrived at our emergency department with sudden onset nontraumatic chest pain for 12 hours accompanied by dyspnea, nausea and vomiting in September 2019. No significant medical history or family history. Auscultation revealed diastolic murmur but weakness of heart sound, and cardiac function was NYHA class IV.

**Fig. 3:**
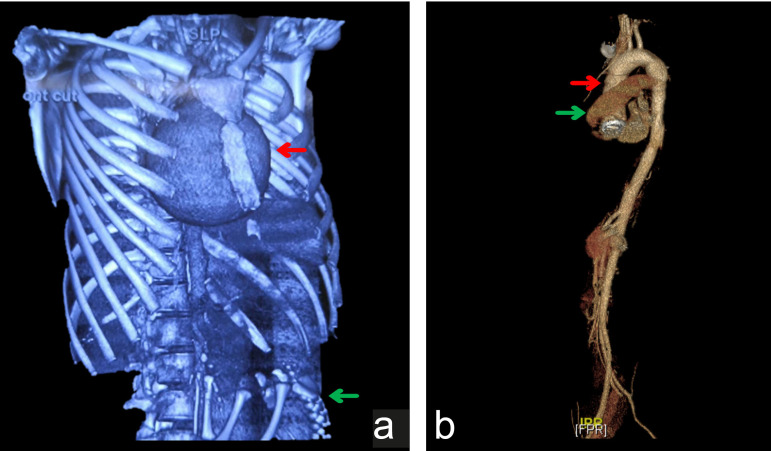
Changes in pre- and postoperative computed tomography angiography images of Case 3. (A) 3D reconstruction of preoperative CT images shows the location of the giant ascending aortic aneurysm marked by a red arrow and the fetal skeleton marked by a green arrow. (B) 3D reconstruction of postoperative CT images shows the artificial blood vessel marked by a red arrow and a circular low-density shadow marked by a green arrow.

In the meantime, no clinical signs of fetal distress were found as the fetal heart rate was 135 beats per minute. Echocardiography revealed an area of aortic valve regurgitation of 23.7 cm^2^, indicating severe AI. FS and LVEF were 33% and 65%, respectively. CT scan revealed that the ascending aorta was enlarged (maximum internal diameter of 101 mm), indicating a giant ascending aortic aneurysm (Figure 3A). Considering the urgency of a symptomatic giant ascending aortic aneurysm and the fetus was clearly viable, an MDT was promptly formed. The patient and her family agreed with surgical repair of the aorta and cesarean delivery after thorough communication, and a written informed consent was obtained.

The patient was escorted to the operating room under invasive monitoring 6.4 hours after arrival. The staged surgical plan consisted of cesarean section, intrauterine balloon tamponade and Bentall procedure. The anesthetic procedure and agents employed were similar to those in Case 1. The balloon and intravenous oxytocin were employed in prevention of postpartum hemorrhage after cesarean section. Apgar scores of the newborn were 7, 10, and 10 at 1, 2 and 10 minutes, respectively. The right coronary artery abnormally originated from the left coronary sinus, according to operative exploration. However, her heart rate dropped abruptly after Bentall procedure. Transesophageal echocardiography revealed that the right coronary artery was significantly compressed. Therefore, coronary artery bypass grafting (CABG) using grafts harvested from the right great saphenous vein was performed after the bypass was re-established. Heartbeat returned spontaneously after surgery. The operating time, cardiopulmonary bypass time and aortic clamping time and selective cerebral perfusion time were 11.4 hours, 232 minutes and 100 minutes, respectively. The patient was transferred to the ICU for close monitoring immediately after surgery. The balloon was deflated 19.3 hours after surgery, and the patient was successfully extubated 26.5 hours after surgery. She was transferred from ICU to the general ward 3 days later. A comprehensive examination for connective disorder was advised to determine the cause of the giant ascending aortic aneurysm after consultation with rheumatology experts. Unfortunately, she refused to proceed due to economic reasons. She was discharged home 16 days later, during which no significant complications were observed. To date, she is alive with an estimated NYHA class I. FS and LVEF during follow-up were 33% and 67%, respectively. CTA revealed that the ascending aorta was irregular in shape, surrounded by a circular low-density shadow, but no enhancement was found (Figure 3B). Meanwhile, the baby was in good health.

A summary of the clinical characteristics and treatment outcomes of the 3 cases is presented in Table 1.

**Table 1:** Clinical data of the patients.

Variable	Case 1	Case 2	Case 3
Age (yrs)	30	32	27
BMI (kg/m^2^)	21.9	24.0	23.7
Pregnancy (weeks)	34	37	37
Diameter of ascending aorta (mm)	52	39	102
Aortic insufficiency	Mild-moderate	Mild-moderate	Severe
Preoperative FS (%)	40	41	33
Preoperative LVEF (%)	70	61	65
Marfan syndrome	Y	Not tested	Not tested
Preoperative cardiac function (NYHA)	III	III	IV
Interval between arrival at emergency department and surgery (h)	10.7	5.1	6.4
Intervention	Bentall + TAR + FET + repeat C-section + IUBT + bilateral tubal ligation	Bentall + TAR + FET + repeat C-section + IUBT	Bentall + CABG + temporary pacemaker implantation + cesarean section + IUBT
Cardiac activity	Spontaneous	Electrical cardioversion	Spontaneous
Selective cerebral perfusion (min)	32	35	N
Aortic clamping (min)	178	151	100
Cardiopulmonary bypass time (min)	273	234	232
Operating time (h)	10.4	9.1	11.4
Mechanical ventilation (h)	15.8	20.7	26.5
Intrauterine balloon tamponade (h)	33.8	20.6	19.3
ICU stay(d)	5	3	3
Hospital stay (d)	18	15	16
Hypoxia	Y	N	N
Nerve damage	N	Y	N
Follow-up (d)	751	575	250
Pneumothorax/hydropneumothorax	N	Y	N
Recurrence	N	N	N
Death	N	N	N
FS during follow-up (%)	37	36.6	33
LVEF during follow-up (%)	67	66.3	62
Cardiac function during follow-up (NYHA)	I	II	II

FS=fractional shortening; LVEF=left ventricular ejection fraction; CABG=coronary artery bypass grafting; TAR=total aortic arch replacement; FET=frozen elephant trunk; IUBT=intrauterine balloon tamponade

## DISCUSSION

Aortic dissection during pregnancy is not commonly found in clinical practice, with an incidence of 0.4-0.5 per 100,000. However, it remains the most common major cause of death in pregnant women suffering from cardiovascular disease, as mortality from this condition is as high as 60%^[5-7]^. Stanford type A aortic dissection is most commonly found in this situation. Women will experience significant changes in hemodynamics and hematology during pregnancy, such as accelerated heart rate, increased cardiac output, increased left ventricular thickness, etc.^[8]^. Plasma volume and overall erythrocyte weight will increase by 45% and 20% during late pregnancy^[9]^. Meanwhile, abdominal aorta and iliac artery will be compressed by the enlarged uterus, resulting in pregnancy-induced hypertension^[10]^. Besides, the increased secretion of estrogen and progesterone during pregnancy will lead to significant changes in the structures of aortic wall, such as destruction of elastic laminae, decreased proteoglycan, hypertrophy and hyperplasia of vascular smooth muscle, etc.^[11]^. These changes would be amplified in pregnant women suffering from hypertension, pre-eclampsia or potential aortic disease (such as Marfan syndrome), making them much more vulnerable to aortic dissection^[12]^.

In this report, we present three cases of late pregnancy complicated by AAAD, all diagnosed and managed by our MDT. With the help of multidisciplinary cooperation, all patients recovered well and were discharged home after surgical intervention composed of aorta repair and cesarean delivery in a timely and proper fashion. To date, patients and their babies are healthy without any further complication. The main complaints in pregnant women with AAD were sudden-onset continuous tearing-like chest and back pain, which cannot be relieved, with or without dyspnea^[13]^. In compliance to our government policy, a chest pain center has been established in our emergency department since 2011, which provides a “green channel” and a well-established system for all patients with chest pain. An MDT is on call at any time to deal with the complex cases in our center. A standard procedure is followed and an MDT is formed when pregnant women with symptoms mentioned above presented to our center. Briefly, bedside cardiac echocardiography and CTA for thoracic and abdominal aorta are urgently performed by medical imaging team, since both modalities can provide pivotal information in discussion of aortic diagnosis^[14]^. As recommended, AAAD should be managed as urgently as possible, once a diagnosis was established via imaging, because mortality in patients with this condition would rise at a rate of 1%-2% per hour^[15,16]^. In order to make every second count, experts from cardiovascular surgery, intensive care, anesthesia, pediatrics and obstetrics are urgently brought together to orchestrate an optimal plan for the patient, while experts in our patient communication unit communicate with the patient and her family comprehensively and promptly to make sure that they fully understand the patient’s condition, the intervention plan and the risk of surgery. Pregnant women with other risk factors for aortic dissection, such as Marfan syndrome, Turner syndrome and/or bicuspid aortic valve, are treated with higher priority and proper medical guidance^[17]^.

The MDT should weigh the balance between severity of the patient’s condition and maturity of the fetus when considering the choice between a conserved plan and an invasive plan for pregnant women with AAD. The well-being of the mother, however, should always be the priority in life-threatening situations. Surgical intervention in this condition should be planned to ensure the safety of the mother because the fetal outcome largely depends on maternal well-being^[18]^. Additionally, cesarean delivery before surgical repair of aortic defect may be a better risk-benefit ratio in pregnant women with AAD if the fetus is viable, because fetal mortality was much higher in pregnant women with AAAD who underwent surgical repair of aortic defect but not cesarean section according to previous reports^[19,20]^. In this report, all three patients with late pregnancy complicated by AAD underwent cesarean section followed by surgical repair of the aortic defect. Besides, patients 1 and 2 underwent aortic valve replacement to decrease the risk of reoperation, even if they had only mild to moderate AI.

A reasonable decision on anesthesia is crucial for the best outcomes of the fetus and the mother. Remifentanil, an ultra-short acting opioid with a short half-life and rapid elimination, is capable of crossing the placental barrier. Therefore, it would not significantly affect the Apgar scores at 5 minutes after delivery and thereafter, since it would be rapidly eliminated in fetus when administered in pregnant women with AAD^[21,22]^. Meanwhile, a reasonable degree of anesthesia is another key to a successful intervention. Anesthetic overdose would result in intrauterine distress, while anesthetic underdose would lead to aortic dissection due to stimulation from the cesarean section. Therefore, intrauterine hypoxia is closely monitored by an antepartum fetal monitor, with a team of anesthesiology, pediatrics and obstetrics specialists throughout the procedure.

Systemic heparinization is essential for establishing cardiopulmonary bypass during cardiac surgery. In a patient undergoing cesarean section followed by cardiac surgery, however, systemic heparinization may place the patient at risk for massive atonic postpartum hemorrhage. Hysterectomy can effectively decrease hemorrhage according to the experience from a single center^[23]^. However, a cautious approach should be taken when considering the need for hysterectomy according to our center’s experience, because hysterectomy may accelerate premature ovarian failure while patients and their families may want to preserve fertility^[24]^. Intrauterine balloon is a commonly used obstetrical equipment to control bleeding by placing a Bakri balloon in the uterus and filling it with saline solution, while it is rarely applied in cardiac surgery^[25]^. In this report, all three patients were successfully treated with intrauterine balloon tamponade and intravenous oxytocin. Bilateral tubal ligation or hysterectomy can be considered if patients have no intention of future pregnancy and/or have hemorrhage not controlled by intrauterine balloon tamponade. Meanwhile, the clinician should remain vigilant for massive postpartum hemorrhage caused by coagulation dysfunction, since coagulation factor is depleted during aortic dissection formation and cardiopulmonary bypass^[26,27]^. Besides, the clinician should also be watchful to intrauterine infection due to continuous intrauterine balloon tamponade. Therefore, in addition to immediate blood infusion and preventive antibiotics, the balloon should be deflated in a timely fashion to prevent intrauterine infection and adhesion.

Del Nido cardioplegic solution can provide protective effect to the myocardium during cardiac surgery in adults, which is no worse than St. Thomas cardioplegia or blood cardioplegia. In the meantime, Del Nido cardioplegic solution may significantly decrease the duration of aortic clamping and cardiopulmonary bypass, because it can provide longer protection for the myocardium in a single perfusion^[28,29]^.

A well-organized pediatric team should be formed throughout the procedure to ensure the best outcome for the baby. Measures should be taken to protect the baby, such as giving steroids to promote fetal lung maturation, infusing magnesium sulfate to protect fetal nerves, etc.^[30,31]^.

Despite the breakthrough in these years, our experience is limited by a small sample, lack of variety of diseases, insufficient follow-up duration, etc. According to a report by Zhu et al.^[32]^, promptly fetal monitoring or artificially induced abortion before emergent repair of aortic dissection were advised for AAD patients with gestation not exceeding 28 weeks, because the most unfavorable outcome in patients and/or fetuses undergoing cardiac surgery during pregnancy have been attributed to the adverse effects of cardiopulmonary bypass^[33]^. On the other hand, the maternal well-being should be taken as the priority for the AAD patient with a gestation over 28 weeks. The optimal intervention for both mother and baby may be an emergency cesarean section followed by surgical repair of the aortic defect. Li et al.^[34]^ have shown that blocking the proximal descending aorta with a balloon after incision of the aortic arch and maintaining circulation through the femoral artery can significantly decrease ischemia duration and postoperative complications if AAAD involves the aortic arch or even the descending aorta. As for pregnancy complicated by type B AAD, the best choice is medical therapy or thoracic endovascular aortic repair, unless complicated by another condition that requires open surgery, such as rupture of aortic dissection.

## CONCLUSION

In conclusion, we employed a multidisciplinary approach in managing three cases of late pregnancy complicated by AAAD. All patients survived without serious complications during the follow-up. Therefore, multidisciplinary cooperation is essential in the diagnosis of the pregnant patient with AAD and in the orchestration of individual therapy, which is essential for the well-being of both the mother and the baby.

### Ethic Statement

The authors are accountable for all aspects of the work in ensuring that questions related to the accuracy or integrity of any part of the work are appropriately investigated and resolved.

## References

[1] Hagan PG, Nienaber CA, Isselbacher EM, Bruckman D, Karavite DJ, Russman PL (2000). The International Registry of Acute Aortic Dissection (IRAD): new
insights into an old disease. JAMA.

[2] Sawlani N, Shroff A, Vidovich MI (2015). Aortic dissection and mortality associated with pregnancy in the
United States. J Am Coll Cardiol.

[3] Ch'ng SL, Cochrane AD, Goldstein J, Smith JA (2013). Stanford type a aortic dissection in pregnancy: a diagnostic and
management challenge. Heart Lung Circ.

[4] Kamel H, Roman MJ, Pitcher A, Devereux RB (2016). Pregnancy and the Risk of Aortic Dissection or Rupture: A
Cohort-Crossover Analysis. Circulation.

[5] Smith K, Gros B (2017). Pregnancy-related acute aortic dissection in Marfan syndrome: A
review of the literature. Congenit Heart Dis.

[6] Wilkinson H, Trustees and Medical Advisers (2011). Saving mothers' lives. Reviewing maternal deaths to make
motherhood safer: 2006-2008. BJOG.

[7] Immer FF, Bansi AG, Immer-Bansi AS, McDougall J, Zehr KJ, Schaff HV, Carrel TP (2003). Aortic dissection in pregnancy: analysis of risk factors and
outcome. Ann Thorac Surg.

[8] Smok DA (2014). Aortopathy in pregnancy. Semin Perinatol.

[9] Ramlakhan KP, Johnson MR, Roos-Hesselink JW (2020). Pregnancy and cardiovascular disease. Nat Rev Cardiol.

[10] Crawford JD, Hsieh CM, Schenning RC, Slater MS, Landry GJ, Moneta GL, Mitchell EL (2016). Genetics, Pregnancy, and Aortic Degeneration. Ann Vasc Surg.

[11] Manalo-Estrella P, Barker AE (1967). Histopathologic findings in human aortic media associated with
pregnancy. Arch Pathol.

[12] Meijboom LJ, Vos FE, Timmermans J, Boers GH, Zwinderman AH, Mulder BJ (2005). Pregnancy and aortic root growth in the Marfan syndrome: a
prospective study. Eur Heart J.

[13] Yang G, Peng W, Zhao Q, Peng J, Xiang X, Chai X (2015). Aortic dissection in women during the course of pregnancy or
puerperium: a report of 11 cases in central south China. Int J Clin Exp Med.

[14] Bossone E, LaBounty TM, Eagle KA (2018). Acute aortic syndromes: diagnosis and management, an
update. Eur Heart J.

[15] Patel C, Akhtar H, Gupta S, Harky A (2020). Pregnancy and cardiac interventions: What are the optimal
management options?. J Card Surg.

[16] Hiraya D, Sato A, Aonuma K (2018). Circulating microRNAs as an emerging biomarker for acute aortic
dissection diagnosis-comparing with prior biomarkers. J Thorac Dis.

[17] Rajagopalan S, Nwazota N, Chandrasekhar S (2014). Outcomes in pregnant women with acute aortic dissections: a
review of the literature from 2003 to 2013. Int J Obstet Anesth.

[18] van Hagen IM, Cornette J, Johnson MR, Roos-Hesselink JW. (2017). Managing cardiac emergencies in pregnancy. Heart.

[19] Liu Y, Han F, Zhuang J, Liu X, Chen J, Huang H (2020). Cardiac operation under cardiopulmonary bypass during
pregnancy. J Cardiothorac Surg.

[20] Regitz-Zagrosek V, Blomstrom Lundqvist C, Borghi C, European Society of Gynecology (ESG); Association for European
Paediatric Cardiology (AEPC); German Society for Gender Medicine
(DGesGM) (2011). ESC Guidelines on the management of cardiovascular diseases
during pregnancy: the Task Force on the Management of Cardiovascular
Diseases during Pregnancy of the European Society of Cardiology
(ESC). Eur Heart J.

[21] Bingel U, Wanigasekera V, Wiech K, Ni Mhuircheartaigh R, Lee MC, Ploner M (2011). The effect of treatment expectation on drug efficacy: imaging the
analgesic benefit of the opioid remifentanil. Sci Transl Med.

[22] Noskova P, Blaha J, Bakhouche H, Kubatova J, Ulrichova J, Marusicova P (2015). Neonatal effect of remifentanil in general anaesthesia for
caesarean section: a randomized trial. BMC Anesthesiol.

[23] Kuroda Y, Uchida T, Hamasaki A, Yamashita A, Mizumoto M, Akabane K (2019). Surgery for Acute Type A Aortic Dissection in A Pregnant Woman At
28 Weeks' Gestation. Braz J Cardiovasc Surg.

[24] Khadilkar S (2020). Does Saving Uterus Save Ovaries?. J Obstet Gynaecol India.

[25] Quandalle A, Ghesquière L, Kyheng M, Ducloy AS, Subtil D, Debarge V (2021). Impact of intrauterine balloon tamponade on emergency peripartum
hysterectomy following vaginal delivery. Eur J Obstet Gynecol Reprod Biol.

[26] Paparella D, Rotunno C, Guida P, Malvindi PG, Scrascia G, De Palo M (2011). Hemostasis alterations in patients with acute aortic
dissection. Ann Thorac Surg.

[27] Guan XL, Wang XL, Liu YY, Lan F, Gong M, Li HY (2016). Changes in the Hemostatic System of Patients With Acute Aortic
Dissection Undergoing Aortic Arch Surgery. Ann Thorac Surg.

[28] Ler A, Sazzad F, Ong GS, Kofidis T (2020). Comparison of outcomes of the use of Del Nido and St. Thomas
cardioplegia in adult and paediatric cardiac surgery: a systematic review
and meta-analysis. Perfusion.

[29] Kuserli Y, Turkyilmaz S, Turkyilmaz G, Kavala AA (2020). Comparison of del Nido Cardioplegia and Blood Cardioplegia in
Aortic Root Surgery. Heart Surg Forum.

[30] Schmidt AF, Kemp MW, Rittenschober-Böhm J, Kannan PS, Usuda H, Saito M (2018). Low-dose betamethasone-acetate for fetal lung maturation in
preterm sheep. Am J Obstet Gynecol.

[31] Shaw O, Yager JY (2019). Preventing childhood and lifelong disability: Maternal dietary
supplementation for perinatal brain injury. Pharmacol Res.

[32] Zhu JM, Ma WG, Peterss S, Wang LF, Qiao ZY, Ziganshin BA (2017). Aortic Dissection in Pregnancy: Management Strategy and
Outcomes. Ann Thorac Surg.

[33] Kapoor MC. (2014). Cardiopulmonary bypass in pregnancy. Ann Card Anaesth.

[34] Li X, Zhang HY, Han FZ, Yu CJ, Fan XP, Fan RX (2017). [Surgical management of pregnancy-associated acute Stanford type
A aortic dissection: analysis of 5 cases]. Nan Fang Yi Ke Da Xue Xue Bao.

